# Supplementary material for: Investigating the influence of physiologically relevant hydrostatic pressure on CHO cell batch culture
Source: Sci Rep. 2021 Jan 8;11:162. doi: 10.1038/s41598-020-80576-8 (PMC7794228; doi:10.1038/s41598-020-80576-8)
Supplement: Supplementary file 1 — Supplementary Information 1. [file 41598_2020_80576_MOESM1_ESM.zip › S1/IgG/Feb14 0 30 60 90/Blank.pdf]

Data File C:\Users\P...s\ChemStation\1\Data\TK_CHO_IgG 2019-02-14 10-52-34\001-1-OPTICHO.D
Sample Name: OPTICHO

===

Acq. Operator : SYSTEM   Seq. Line : 1
Sample Operator : SYSTEM
Acq. Instrument : HPLC-0039   Location : 1
Injection Date : 2/14/2019 10:54:31 AM   Inj : 1
Inj Volume : 100.000 µl
Different Inj Volume from Sample Entry! Actual Inj Volume : 50.000 µl
Sequence File : C:\Users\Public\Documents\ChemStation\1\Data\TK_CHO_IgG 2019-02-14 10-52-34 \TK_CHO_IgG.S
Method : C:\Users\Public\Documents\ChemStation\1\Data\TK_CHO_IgG 2019-02-14 10-52-34 \CHO_IgG_LC.M (Sequence Method)
Last changed : 2/8/2019 3:17:23 PM by SYSTEM

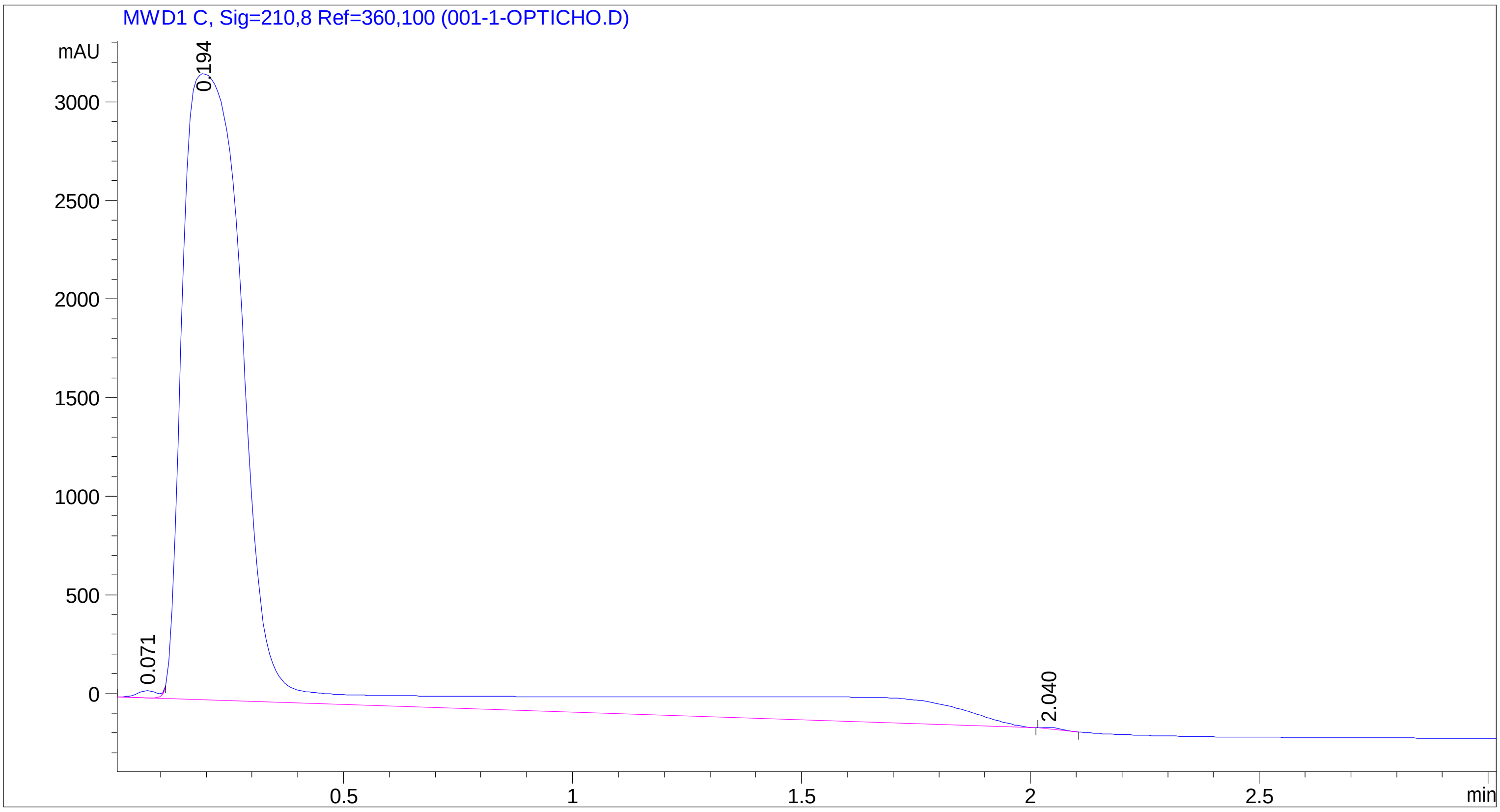

===

Area Percent Report

===

Sorted By : Signal
Multiplier : 1.0000
Dilution : 1.0000
Use Multiplier & Dilution Factor with ISTDs

Signal 1: MWD1 C, Sig=210,8 Ref=360,100

Peak #	RetTime [min]	Type	Width [min]	Area [mAU*s]	Height [mAU]	Area %
1	0.071	BV E	0.0466	107.31100	36.14719	0.2986
2	0.194	VB R	0.1736	3.58069e4	3174.28174	99.6367
3	2.040	BBA	0.0453	23.26275	8.14635	0.0647

Totals : 3.59375e4 3218.57528

===

*** End of Report ***
